# Targeting the Risk of Diptera-Borne Zoonoses by a Sentinel Equestrian Centers Program

**DOI:** 10.3390/pathogens14070661

**Published:** 2025-07-04

**Authors:** Cristiana Cazapal-Monteiro, David Boso, Inês Abreu, Mercedes Camiña, Jaime Sanchís, Adolfo Paz-Silva, Luis Cardoso, Rita Sánchez-Andrade, María Sol Arias, José Ángel Hernández

**Affiliations:** 1 Control of Parasites Research Group (COPAR, GI-2120), Department of Animal Pathology, Faculty of Veterinary, University of Santiago de Compostela, 27002 Lugo, Spain; david.boso@rai.usc.es (D.B.); inesabreu.ramos@usc.es (I.A.); adolfo.paz@usc.es (A.P.-S.); rita.sanchez-andrade@usc.es (R.S.-A.); mariasol.arias@usc.es (M.S.A.); joseangel.malagon@usc.es (J.Á.H.); 2 Department of Physiology, Faculty of Veterinary, University of Santiago de Compostela, 27002 Lugo, Spain; merchi.camina@usc.es; 3 Parasitology and Parasitic Diseases, Faculty of Veterinary, University of “La República” (Regional Litoral Norte), Salto 50000, Uruguay; sanchisjaime@gmail.com; 4 Department of Veterinary Sciences, and Animal and Veterinary Research Centre (CECAV), University of Trás-os-Montes e Alto Douro (UTAD), 5000-801 Vila Real, Portugal; lcardoso@utad.pt

**Keywords:** Culicidae, Diptera-borne diseases, horses, Northwestern Spain, surveillance, zoonoses

## Abstract

Diptera-borne diseases pose a major threat to global health, and their distribution is constantly changing due to climate change, globalization, and environmental changes. To improve the knowledge of dipteran species and their distribution in equine facilities, CDC-UV and oviposition traps were placed, and the dipping technique was performed in 16 equestrian centers of Northwest (NW) Spain (Galicia and Castilla y León Autonomous Communities) between July and November 2023. A questionnaire was distributed among the horse owners to obtain additional information. Four genera of culicids, *Culex* (51.8%), *Culiseta* (38.6%), *Anopheles* (8.4%), and *Aedes*/*Ochlerotatus* (1.2%) were identified in the equestrian centers. *Culex pipiens* s.l. was the most prevalent and well-distributed species (93.8% of the centers), whereas *Anopheles maculipennis* s.l. and *An. claviger*/*petragnani*, the anopheline species, were the most frequent (37.5% and 31.2%, respectively). The *Culiseta* genus was found in approximately 81.2% of the equine facilities. All genera were collected at medium and high altitudes and in Csb (warm-summer Mediterranean climate) areas. Equestrian centers from NW Spain albeit a variety of culicids with high vectorial capacity, together with an ideal environment for their breeding, the presence of vectors and hosts (humans and animals). This potential problem for global health enhances the need for entomological surveillance.

## 1. Introduction

Order Diptera-borne pathogens and their diseases affect more than 300 million people all over the world [1] every year. There are up to 100 pathogens that can be transmitted by female mosquitoes due to their hematophagy behavior, but most of them are limited to tropical and subtropical climates where these vectors are found [2]. In Europe, several species of culicids are widely known for their role in the transmission of different diseases, many of which are endemic in at least one country [3].

The appearance and expansion of vector-borne diseases are due to multifactorial causes, mainly those related to the imbalance of ecosystems and the interaction of pathogens, vectors, and hosts [4,5]. Different countries are registering some changes in climate, mainly involving an incremental increase in temperature [6]. This seems to be related to the fact that, in recent years, new invasive species belonging to the genus *Aedes* (i.e., *Ae. albopictus*, *Ae. aegypti*, *Ae. japonicus*, *Ae. atropalpus*, and *Ae. koreicus*) have been detected, capable of establishing their colonies in certain regions that were previously free of these species [7,8,9].

Spain is considered a country of potential risk for the entry of invasive mosquito species, as well as for the entry and establishment of different emerging pathogens transmitted by them [10,11,12]. At present, Galicia in Northwestern (NW) Spain is not considered an endemic region for any mosquito-borne diseases, but the presence of the three genera from the family Culicidae (i.e., *Culex*, *Anopheles*, and *Aedes*), which may pose a risk to public health, has been described [13]. In particular, several species that can act as vectors of zoonotic pathogens have been identified, such as *Culex pipiens*, *Culex theileri*, *Aedes vexans*, and *Aedes caspius* [14].

**Figure 1:**
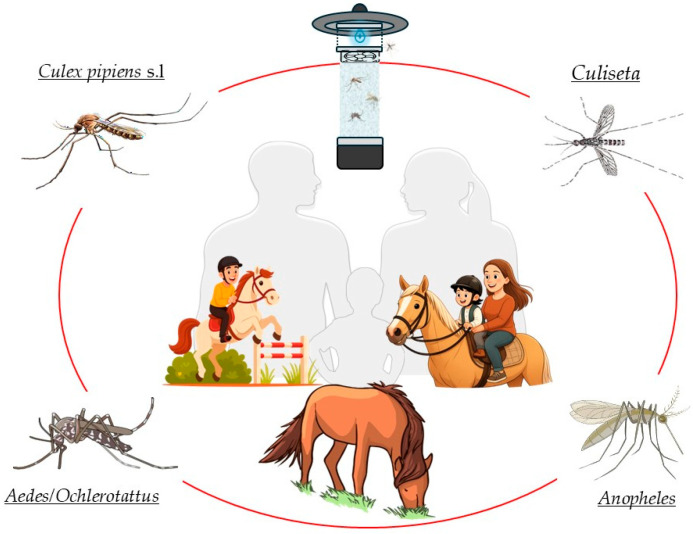
The prevention of Diptera-borne diseases requires surveillance, including those places where people enjoy animals.

An effective surveillance system for human and animal diseases is essential for early detection and rapid response, as well as for prevention and control programs, while allowing for the effectiveness of implemented strategies to be assessed. In this context, it appears essential to obtain information about the dipteran species that might affect certain animal species of particular interest to people, for the purpose of assessing the risk of some diseases that might spread to humans as well (Figure 1). Under this approach, surveillance of facilities containing animals can offer very useful information regarding the potential risk of certain vector-borne diseases. The wide variety of species involved and the often complex natural history of zoonotic agents present a real challenge [15]. This way, the identification of potential risks to public and animal health, as well as the development of control and prevention programs, requires knowledge of the species present in each region in addition to ecological and wildlife studies in reservoirs or vectors [16].

The usefulness of animals as sentinels to monitor certain mosquito-borne zoonoses has been previously stated [11]. In the present study, the value of the surveillance of the main dipteran species in equestrian centers in NW Spain was conducted to assess the risk of possible zoonoses that could be transmitted.

## 2. Materials and Methods

### 2.1. Area of Study

**Figure 2:**
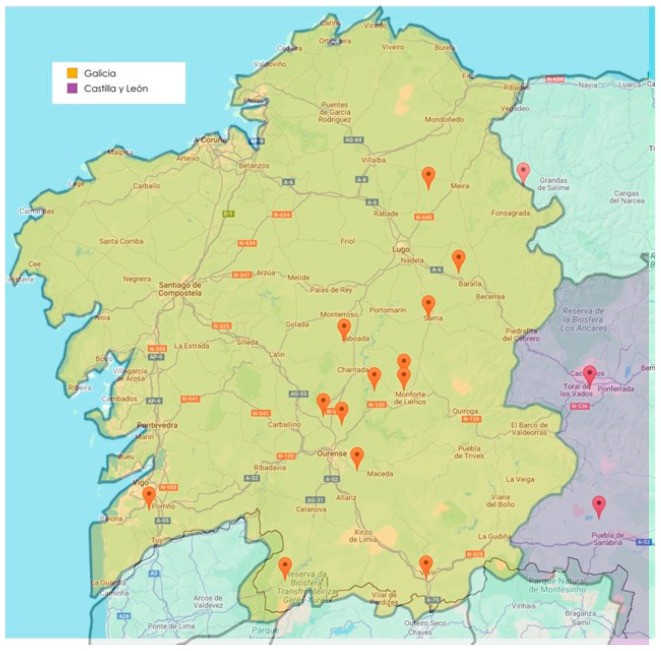
Locations of equine centers involved in the current study. *Map created with EZ Maps and MapChart.*

Between July and November 2023, a total of 16 equestrian centers and stud farms in NW Spain were sampled by placing different trapping devices (Figure 2).

The owners were contacted by phone and all accepted to participate, with the agreement to send them a brief report including the results. The area of the study comprised two autonomous communities, Galicia and Castilla y León. To establish the climatic areas of these regions, the Köppen-Geiger classification was contemplated; hence, two climatic areas usually occurring on the western side of the continent between the latitudes of 30° and 45° were considered [17]:

− Csb or warm-summer Mediterranean climate: characterized by the coldest month averaging above 0 °C, all months with average temperatures below 22 °C, and at least four months averaging above 10 °C.
− Csa or hot-summer Mediterranean climate: Summers are dry and hot, with at least 22 °C averaged across the night–day cycle. The average temperature of the coldest month is <18 °C but above −3 °C. The wettest winter month has about 3 times as much precipitation compared to the driest summer month.

The altitude where the equine centers are located was also considered, then four classes were established according to prior investigations [18]:

− Low: below 300 m above sea level (asl). → → High: between 501 and 1000 m asl.
− Medium: between 301 and 500 m asl. → → Elevated: Above 1000 m asl.

### 2.2. Collection of Diptera

**Figure 3:**
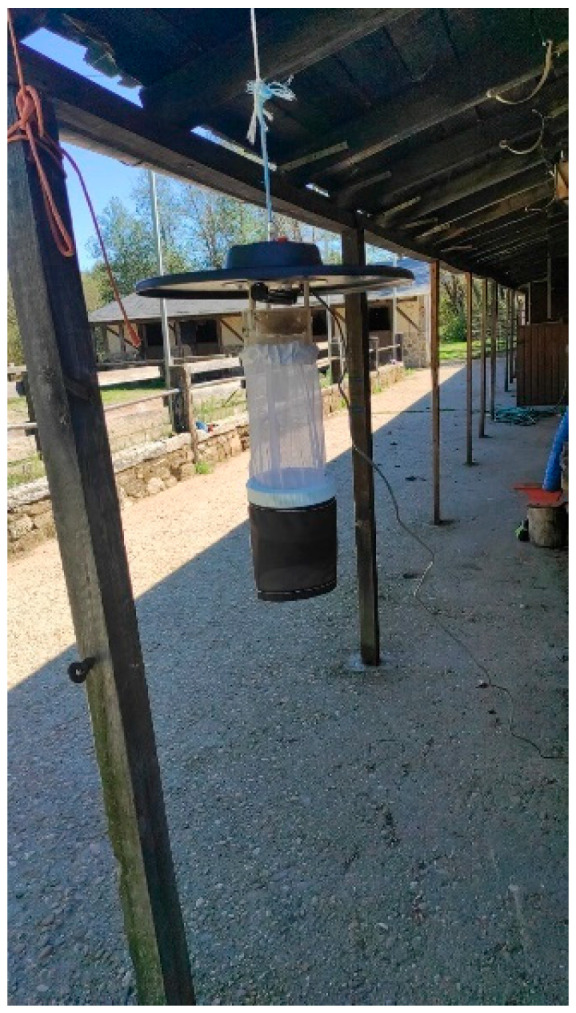
CDC-UV traps placed in equestrian centers to capture dipterans.

A total of 77 samplings were conducted, involving 63 with CDC-UV (ultraviolet) traps, two oviposition traps, and 12 dipping. One miniature downdraft blacklight (UV) trap Model 512 (Entomopraxis^®^, Barcelona, Spain) (Figure 3), which allows for the capture of adult dipterans, was placed in each center close to the animals at 1.7 m above ground and left running continuously from dusk until the following morning (12 h approximately).

The collection tanks were transferred to the COPAR Laboratory (Faculty of Veterinary Medicine of Lugo, University of Santiago de Compostela, Spain) and frozen at −20 °C. Later, the identification was performed under a stereomicroscope (Leica EZ4D, Wetzlar, Germany), by using taxonomic and interactive keys [19,20,21].

Oviposition traps (Entomopraxis^®^, Barcelona, Spain), consisting of a black plastic pot filled with 500 mL of tap water and a small piece of wood leaning on the walls of the container, were left for 15 days in previously chosen locations because of their suitable conditions for culicid breeding (shadowed places). After this period of time, their contents were collected in stagnant polyethylene jars and taken to the same laboratory, where the fourth-stage larvae or even adults were identified, using a taxonomic key [19].

**Figure 4:**
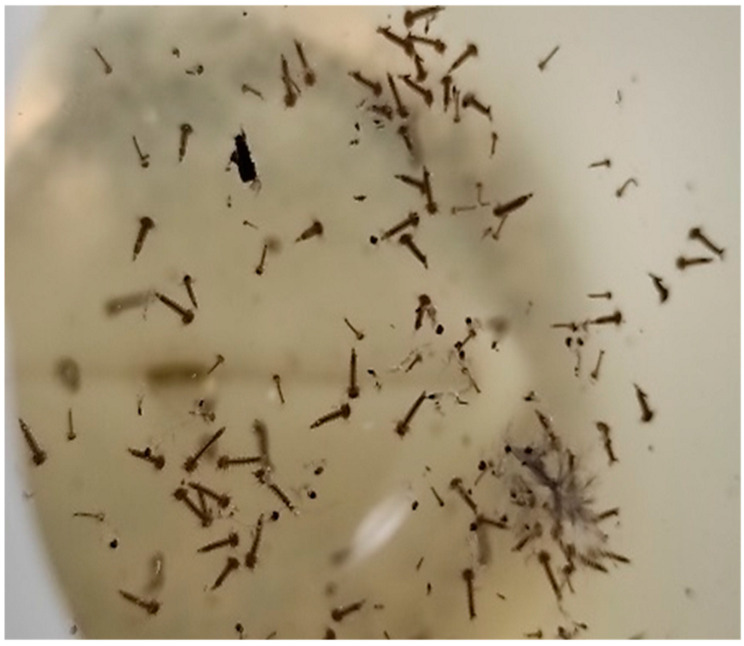
Dipteran larvae collected by oviposition traps.

The dipping technique, an active sampling method that enables the capture of larvae, was carried out in the areas of stagnant water, drinkers, barrels, and tires that were close to the animals or facilities, since those are the breeding areas for culicids. Stagnant water (500 mL) was collected with polyethylene wide-mouth jars (Figure 4) at each sampling and later analyzed in the COPAR laboratory, looking for culicid larvae.

### 2.3. Questionnaire

**Figure 5:**
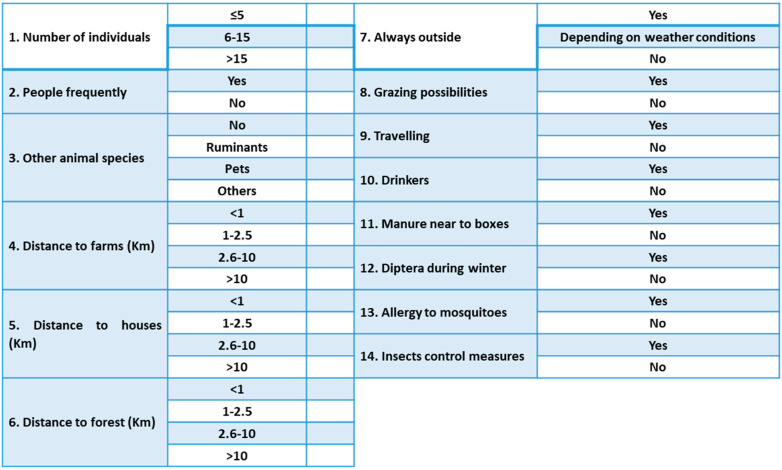
A survey distributed among horse owners to gather information about the main points related to the possible presence of dipterans.

A brief questionnaire was distributed among the owners of the equestrian centers to obtain information on the main points related to the possible presence of dipterans regarding the characteristics of each equine facility, horse management, or insect control measures. Fourteen questions were asked and several answers were possible: number of animals housed, frequency of people present in the facilities, other animal species nearby, distance (km) to farms, houses and forest, stabling regime, grazing possibilities, traveling animals, presence of drinkers in the boxes, presence of manure near to boxes, presence of Diptera during winter, horse allergies to mosquitoes, and insect control measures (Figure 5).

### 2.4. Data Analysis

Data collected were analyzed based on the answers to the survey (Figure 5) and the climatic areas where the equine centers were localized.

Because of data collected in the present investigation were not normally distributed (Kolmogorov–Smirnov test, *p* < 0.05), then the non-parametric Kruskal–Wallis test was applied to compare medians between more two groups or more than two groups, respectively, by using the IBM SPSS statistical package v. 22.0 (Chicago, IL, 139 USA). Significance was considered at *p* < 0.05.

## 3. Results

### 3.1. Specimens Identified

Of a total of 77 samples, 57 were positive (74%). A total of 48/63 (76.2%) captures made with CDC-UV traps and 9/12 (75%) dipping performed were positive, while both oviposition traps remained negative. The specimens collected belonged to the families Culicidae and Ceratopogonidae. Of the Culicidae family, 334 specimens were classified as belonging to genera *Anopheles* (n = 28), Culex (n = 173), *Culiseta* (n = 129), and *Aedes/Ochlerotatus* (n = 4) (Table 1); a total of 282 (84.43%) specimens were collected by CDC-UV traps and 52 (15.57%) by dipping technique.

Genus *Culex* was the predominant one, representing 51.8% of the captures, with three different species identified: *Cx. pipiens* s.l. (58.9% of the genus and 30.5% of the total captures), *Cx. theileri* (9.8% of the genus and 5.1% of the total), and *Cx. perexiguus/univitattus* (with less than 1% within the genus since a single specimen was found throughout the entire study). The remaining 30.6% of specimens belonging to this genus could not be identified due to the loss of body parts and/or morphological characteristics. The second most abundant genus was *Culiseta* (38.6%), with *Cs. annulata* (67.4% of the genus and 26% of the total) and *Cs. longiareolata* (32.6% of the genus and 12.6% of the total).

From the 28 specimens (8.4%) of the genus *Anopheles* captured, 50% were identified as *An. maculipennis* s.l. (4.2% of the total captures), being the most abundant species of this genus; 25% as belonging to the complex *An. claviger/petragnani* (2.1% of the total); and 0.6% as *An. plumbeus*, with only two specimens. As with some *Culex* specimens, five anophelines (17.9% of all anophelines) could not be identified at the species level due to the poor condition of the specimens when the collection buckets were emptied.

Finally, as far as the family Culicidae is concerned, three adult specimens of *Ae. vexans* and one of *Ochlerotatus leucomelas* were identified.

Of the genus *Culicoides*, two species were identified, *C. obsoletus* and *C. punctatus*, although the exact numbers of specimens were not registered.

### 3.2. Results by Centers

The presence of culicids of interest to public health was confirmed in 15 of the 16 (93.75%) equestrian centers participating in this study (Table 2).

The species *Cx. pipiens* were the most prevalent and well distributed, being identified in 93.75% (15/16) of the centers; *Cx. theileri* was identified in 56.25% (9/16) centers; and *Cx. perexiguus/univitattus* was identified in just one of them (6.25%) (Table 3).

Concerning the genus *Anopheles*, *An. maculipennis* and *An. claviger/petragnani* were the species with the greatest frequency, being present in 37.5% (6/16) and 31.25% of the centers, respectively. Finally, *An. plumbeus* was only identified in one out of the 16 (6. 25%) of the centers.

The genus *Aedes/Ochlerotatus* was identified in only two (12.5%) equestrian centers. One specimen of *Ae. vexans* was captured in both centers, while *Oc. leucomelas* specimens were identified in just one of them.

The genus *Culiseta* was present in 13 of 16 (81.25%) centers, and two species were identified: *Cs. annulata* in 7/16 (43.75%) and *Cs. longiareolata* in 6/16 (37.5%).

By considering the simultaneous collection of different genera or species, more than two genera of culicids were identified in 50% (8/16) of the equestrian centers, with the combination of *Culex* and *Anopheles* being the most common. In 12.5% (2/16) of the centers, two species of the same genus were identified, *Cx. pipiens* and *Cx. theileri*, and only in one center, the three genera, *Culex*, *Anopheles*, and *Aedes*, were detected. On the remaining horse farms, in 25% (4/16) only, *Cx. pipiens* was identified.

In addition, the presence of the family Ceratopogonidae was confirmed in 5/16 (31.25%) equestrian centers by the identification of *C. obsoletus* and *C. punctatus*.

### 3.3. Climatic Areas

According to the climatic areas, 62.5% (10/16) of the equine centers are located in Csb zones and 37.5% in Csa (Table 4).

All four culicid genera, *Anopheles*, *Culex*, *Aedes/Ochlerotatus*, and *Culiseta*, were identified during our study in Csb climatic areas, while *Aedes/Ochlerotatus* was not collected in the Csa zones. The number of *Anopheles* specimens was significantly higher in Csb regions, and *Culex* in Csa. No statistical significance for *Aedes/Ochlerotatus* or *Culiseta*. Although *Culicoides* specimens were identified in both Csb and Csa climatic regions, no statistical association was found.

Regarding the altitude of the equestrian centers’ locations, all dipteran families and genera identified in this study were present at medium and high altitudes (301–1000 m asl) (Table 4), and only *Aedes/Ochlerotatus* was absent at lower and elevated altitudes.

### 3.4. Survey Data

As summarized in Table 5, most of the equine centers visited had more than six horses. Despite the fact that the highest prevalences were observed in those centers with 6–15 individuals, statistically significant differences were not obtained (*p* > 0.05).

Almost half of the centers are dedicated to activities that include teaching horse riding or horseback riding on country trails and in forests, so people are present nearly every day. The highest prevalence values of the genera *Anopheles*, *Culex*, *Culiseta*, and *Culicoides* were recorded in these centers with people, but significant differences were demonstrated only for *Anopheles* (χ^2^ = 6.125, *p* = 0.013) and *Culiseta* (χ^2^ = 5.657, *p* = 0.017) (Table 5).

No differences were observed according to the presence of other animal species, such as pets (dogs and cats). Although most of the centers were 1–10 km away from livestock farms (Table 5), significant differences were not recorded. Most of the equine centers were located within a 2.5 km distance to housing or forested areas, but no statistically significant differences were found.

Concerning the keeping conditions, half of the centers always kept the horses outside where they can graze during the day, and despite the highest prevalence values of Diptera being achieved in these centers, no significant influence was observed (Table 5). A similar pattern was obtained for those centers where horses travel regularly to participate in meetings, horse races, and other competitions, but significant differences for *Culex* were obtained (χ^2^ = 4.468, *p* = 0.031).

**Figure 6:**
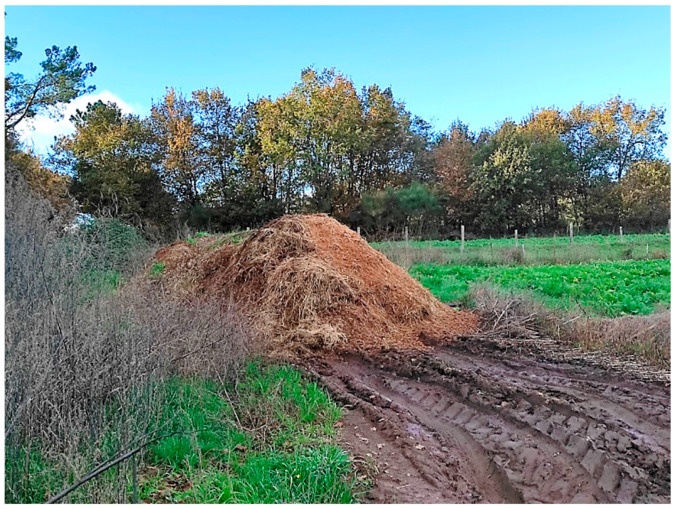
Piles of manure stored close to the facilities.

In most equine centers, there are automated horse waterers, and the presence of *Anopheles* was significantly lower in these facilities (Table 5). In half of the centers where manure was stored next to the buildings (0–5 m), the highest prevalence of Diptera was found (*p* > 0.05) (Figure 6).

The presence of Diptera during winter was detected in 37.5% of the centers. Horse allergies to mosquitoes were reported in 31.25% of the centers, and significant differences were demonstrated in the prevalence of *Culiseta* (χ^2^ = 5.303, *p* = 0.021) (Table 5). Finally, only two equine centers applied some measures for the control of insects, consisting of fly repellents and Asian hornet (*Vespa velutina*) traps.

## 4. Discussion

The surveillance of dipterans in equestrian centers from NW Spain revealed the presence of culicid genera in all of them, and except for one, the existence of species of sanitary interest due to their role as vectors of various diseases was confirmed. The predominant genus was *Culex*, the most abundant and widely distributed in Galicia [13]. The species *Cx. pipiens* s.l. was the most frequent, in agreement with previous investigations conducted in the same area [14,22,23] and on equine farms in other European areas, such as Belgium [24]. Based on its high ability to adapt to different habitats and little specificity for its hosts, several generations can occur per year [25]. The importance of *Cx. pipiens* s.l. lies in its great vectorial capacity for various zoonotic pathogens, playing an important role in the transmission of different arboviruses (e.g., West Nile virus and Usutu virus), as well as the parasitic nematode *Dirofilaria immitis*; therefore, it has acquired great relevance in public and animal health [26]. Two other genera of culicids with importance for public health have also been detected, such as *Anopheles* and *Aedes*/*Ochlerotatus*.

In the current study, three species/complexes belonging to the genus *Anopheles* were identified, *An. maculipennis* s.l., *An. Plumbeus*, and *An. claviger/petragnani*. This genus is widely distributed in the Iberian Peninsula and has a preference for natural areas with abundant vegetation, near permanent natural water bodies rich in organic matter [14], conditions which were frequently observed in the current study. Although the presence of *Anopheles* was significantly higher in Csb climatic areas (warm-summer Mediterranean), it was also identified in Csa (hot-summer Mediterranean) zones. These results highlight that this genus has a wide distribution in Galicia. Regarding the genus *Aedes*, two specimens of *Ae. vexans* were identified in Csb areas, a situation which agrees with previous descriptions [27]. The genus *Culiseta* was widely distributed among the equestrian centers, thus confirming their huge circulation in Galicia [28]. *Culiseta longiareolata* can be a vector of avian paludism but has no implications for public health, while *Cs. annulata* is involved in the transmission of Tahyna virus to humans, avian paludism, and rabbit myxomatosis [29]. The family Ceratopogonidae was detected in almost one-third of the centers in both Csb and Csa climatic areas, also confirming prior findings [13,27,28]. This genus has great importance for animal health since its representatives are vectors of serious animal diseases as Bluetongue and epizootic hemorrhagic disease, affecting domestic and wild ruminants [30], and African horse sickness (AHS), which, despite not being present in Europe, has been responsible for major outbreaks originated by the introduction of reservoirs from endemic areas (Africa) in various European countries, including Spain. Furthermore, due to its high lethality and the severity of the clinical conditions that occur in horses, AHS is a disease [31].

Results presented here, where ten species of culicids were identified, underline the important diversity of dipteran species, which might act as vectors of several pathogens, in equestrian centers from NW Spain. Other associated issues include disturbances to people and animals being bitten by them, a circumstance which frequently results in a substantial local inflammatory response [32]. The lack of differences in the presence of dipterans regarding the horses’ keeping conditions, especially during their continuous maintenance under field conditions, appears to indicate that dipterans are attracted to stables or boxes, which might explain why a little more than one-third of horse owners observed them during winter. Breeding areas are different for both dipteran families. While culicids are strictly aquatic and lay their eggs in natural or artificial stagnant waters found near hosts, *Culicoides* require organic matter and humidity, such as feces or manure stored near animals [33]. In the same way, no influence was recorded on the distance between the horse centers and the localization of houses or forested zones, a fact that reflects the wide distribution of dipterans in the study area.

Diptera-borne pathogens and the diseases they cause are emerging and re-emerging in Europe, mainly due to climate change, globalization, and environmental changes, natural or artificial, and many of them are zoonotic in nature with a great impact on public health [4,9]. At the same time, these diseases are mostly influenced by climate change since these vectors are highly dependent on the ecology of the environment, and so the weather conditions will determine the presence and distribution of mosquitoes [34]. The presence of vector-borne diseases will always depend on the coexistence of suitable hosts, vectors, and pathogens [35]. As with other entertainment facilities, equine centers provide considerable benefits for people to enjoy nature, relaxation, or walks. These facilities can also ensure the conditions to enhance the presence of different genera of culicids with great vectorial capacity, as demonstrated in more than half of the equine centers in the present investigation. In addition to this, it looks very conceivable that the constant presence of animals (horses) and humans (horse riders in some cases) provides an ideal environment for maintaining the cycle of different species of hematophagous dipterans as they can serve as potential hosts for the blood meal intake [24,36]. The several species found in this study agree with previous findings in the same area [13,14,27,37].

There is a limited number of zoonoses that can be transmitted between horses and humans under natural circumstances, i.e., Hendra virus, USUTV, or WNV [38], any of which have been reported in Galicia. Although many Diptera-borne zoonotic agents are not yet endemic or established in Europe, such as Dengue, Zyka, Eastern Equine Encephalitis or Saint Louis Encephalitis, it should be emphasized that some equestrian centers house individuals that travel outside the region (250–800 km southward), where they meet other horses of different origins, which may, therefore, pose a risk of exposure to pathogens unidentified in their region or country of origin, thus acting as a gateway to a new territory [15]. Eventually, if a pathogen is introduced, all the conditions are met for its circulation to be established and the disease to appear. In this case, periodical analyses of exposure to certain pathogens seem very useful among traveling horses to avoid the spread and thus, are strongly recommended.

Finally, it is interesting to note that the equine centers in the present research were located in two autonomous communities (Galicia and Castilla-León), with different organizations for the surveillance of diseases, which could make it difficult to apply proper measures for the control of dipterans.

This preliminary study provides very interesting and practical information about the presence of dipterans in equine facilities. More efforts are required to confirm the dipteran species and the possibilities of mosquito-borne diseases, which will cause a great impact on public and veterinary health. To this end, it would be of great interest to extend the sampling season throughout the year to establish its phenology and, also, to extend it geographically to cover the entire community of Galicia.

## 5. Conclusions

Equestrian centers provide suitable conditions for dipteran survival and multiplication, making them proper locations for their surveillance as their presence was confirmed in 93.75% of the sampled equestrian centers. Since some dipterans may be involved in the transmission of numerous diseases, including certain zoonoses, their monitoring is strongly advised. Further studies are in progress to determine the possibility that some species of dipterans may survive overwintering indoors (boxes).

## Figures and Tables

**Table 1:** Dipteran specimens collected in equestrian centers.

Genera	Species	No. of Specimens	Total %	Sum of Percentages
*Anopheles*	*Anopheles* spp.	5	1.5	8.4
	*An. maculipennis*	14	4.2	
	*An. claviger*/*petragnani*	7	2.1	
	*An. plumbeus*	2	0.6	
*Culex*	*Culex* spp.	53	15.9	51.8
	*Cx. pipiens*	102	30.5	
	*Cx. theileri*	17	5.1	
	*Cx. perexiguus/univitattus*	1	0.3	
*Culiseta*	*Cs. annulata*	87	26	38.6
	*Cs. longiareolata*	42	12.6	
*Aedes/Ochlerotatus*	*Ae. vexans*	3	0.9	1.2
	*Oc. leucomelas*	1	0.3	
Total		334	100	100

**Table 2:** The distribution of diptera captured according to the equestrian center and type of climate.

Center	Clime Type	Species/Genera
1	Csb	*Cx. pipiens* s.l. *Cx. theileri*
2	Csb	*Cx. pipiens* s.l. *Cx. theileri* *Culicoides*
3	Csb	*Cx. pipiens* s.l. *Cx. theileri* *An. maculipennis* *Cs. longiareolata* *Cs. annulata*
4	Csa	*Cx. pipiens* s.l. *Cx. theileri* *Cx. perexigus* *An. maculipennis* *Cs. longiareolata* *Culicoides*
5	Csa	*Cx. pipiens* s.l. *Cx. theileri* *An. maculipennis* *An. claviger/petragnani* *Cs. longiareolata* *Cs. annulata*
6	Csb	*Cx. pipiens* s.l.
7	Csb	*Cx. pipiens* s.l. *Cx. theileri* *An. claviger/petragnani* *Cs. longiareolata*
8	Csb	*Cx. pipiens* s.l. *Ae. vexans*
9	Csb	*Cx. pipiens* s.l.
10	Csb	*Cx. pipiens* s.l. *Cx. theileri* *An. maculipennis* *An. claviger/petragnani* *Ae. vexans* *Oc. leucomelas* *Cs. longiareolata* *Cs. annulata*
11	Csa	*Cx. pipiens* s.l. *An. claviger/petragnani* *Cs. annulata* *Culicoides*
12	Csa	*Cx. pipiens* s.l. *Cx. theileri* *An. maculipennis* *An. claviger/petragnani* *An. plumbeus* *Cs. longiareolata* *Cs. annulata* *Culicoides*
13	Csa	*Cx. pipiens* s.l. *Cx. theileri* *An. maculipennis* *Cs. annulata*
14	Csa	*Cx. pipiens* s.l.
15	Csb	*Cx. pipiens* s.l. *Cs. annulata* *Culicoides*
16	Csb	-

**Table 3:** Prevalence (%) of dipteran species captured in equestrian centers.

Family	Genera	Species	% Centers
Culicidae	*Culex*	*Cx. pipiens* s.l.	93.75
		*Cx. theileri*	56.25
		*Cx. perexiguus/univitattus*	6.25
	*Anopheles*	*An. maculipennis* s.l.	37.50
		*An. claviger/petragnani*	31.25
		*An. plumbeus*	6.25
	*Aedes/Ochlerotatus*	*Ae. vexans*	12.50
		*Oc. leucomelas*	6.25
	*Culiseta*	*Cs. longiareolata*	43.75
		*Cs. annulata*	37.50
Ceratopogonidae	*Culicoides*	*data*	31.25

**Table 4:** Diptera genera identified according to the climatic areas and altitude where the equestrian centers are located.

Factor	Diptera Genera				
Climatic area	*Anopheles*	*Culex*	*Aedes/Ochlerotatus*	*Culiseta*	*Culicoides*
Csb (n = 10)	3	9	2	4	2
Csa (n = 6)	5	6	0	5	3
	χ^2^ = 4.021 *p* = 0.045 *	χ^2^ = 1.030 *p* = 0.310	χ^2^ = 1.280 *p* = 0.258	χ^2^ = 1.939 *p* = 0.164	χ^2^ = 1.473 *p* = 0.225
Altitude (m)					
<300 (n = 2)	2	2	0	2	1
301–500 (n = 8)	4	7	1	4	1
501–1000 (n = 5)	2	5	1	3	3
>1000 (n = 1)	0	1	0	0	0
	χ^2^ = 3.272 *p* = 0.352	χ^2^ = 1.2008 *p* = 0.751	χ^2^ = 0.571 *p* = 0.887	χ^2^ = 2.662 *p* = 0.447	χ^2^ = 3.764 *p* = 0.288

* Statistically significant (*p* ≤ 0.05).

**Table 5:** Risk factors for the presence of diptera in equestrian centers according to owners’ responses to the questionnaire.

Factor	Number of Equine Centers Positive to Dipterans (% Within Genus)				
1. Number of horses/center (*n* = number of centers)	*Anopheles*	*Culex*	*Aedes/Ochlerotatus*	*Culiseta*	*Culicoides*
≤5 (n = 4)	1 (12.5)	4 (26.7)	1 (50)	1 (11.1)	0
6–15 (n = 5)	2 (25)	4 (26.7)	1 (50)	2 (22.2)	1 (20)
>15 (n = 7)	5 (62.5)	7 (46.6)	0	6 (66.7)	4 (80)
*Statistics*	χ^2^ = 1.270 *p* = 0.530	χ^2^ = 1.500 *p* = 0.472	χ^2^ = 1.668 *p* = 0.434	χ^2^ = 2.389 *p* = 0.303	χ^2^ = 4.029 *p* = 0.133
2. Frequent presence of people (riding school, horseback riding)					
Yes (n = 9)	7 (87.5)	9 (60)	1 (50)	8 (88.9)	4 (80)
No (n = 7)	1 (12.5)	6 (40)	1 (50)	1 (11.1)	1 (20)
*Statistics*	χ^2^ = 6.125 *p* = 0.013 *	χ^2^ = 1.497 *p* = 0.221	χ^2^ = 0.008 *p* = 0.927	χ^2^ = 5.657 *p* = 0.017 *	χ^2^ = 1.563 *p* = 0.211
3. Other animal species in the center					
No (n = 8)	3 (37.5)	8 (53.3)	2 (100)	4 (44.4)	1 (20)
Ruminants					
Pets (n = 8)	5 (62.5)	7 (46.7)	0	5 (55.6)	4 (80)
Others					
*Statistics*	χ^2^ = 1.507 *p* = 0.304	χ^2^ = 1.618 *p* = 0.203	χ^2^ = 2.133 *p* = 0.144	χ^2^ = 0.710 *p* = 0.399	χ^2^ = 2.455 *p* = 0.117
4. Distance to farms (km)					
<1 (n = 1)	0	1 (6.7)	0	0	1 (20)
1–2.5 (n = 3)	1 (12.5)	3 (20)	1 (50)	1 (11.1)	1 (20)
2.6–10 (n = 6)	5 (62.5)	6 (40)	0	6 (66.7)	3 (60)
>10 (n = 6)	2 (25)	5 (33.3)	1 (50)	2 (22.2)	0
*Statistics*	χ^2^ = 4.363 *p* = 0.225	χ^2^ = 3.001 *p* = 0.391	χ^2^ = 1.972 *p* = 0.578	χ^2^ = 7.159 *p* = 0.067	χ^2^ = 5.545 *p* = 0.136
5. Distance to houses (km)					
<1 (n = 9)	4 (50)	9 (60)	1 (50)	4 (44.4)	2 (40)
1–2.5 (n= 5)	3 (37.5)	4 (26.7)	0	3 (33.3)	2 (40)
2.6–10 (n= 2)	1 (12.5)	2 (13.3)	1 (50)	2 (22.2)	1 (20)
>10					
*Statistics*	χ^2^ = 0.343 *p* = 0.842	χ^2^ = 0.508 *p* = 0.776	χ^2^ = 3.517 *p* = 0.172	χ^2^ = 1.736 *p* = 0.420	χ^2^ = 0.794 *p* = 0.672
6. Distance to forest (km)					
<1 (n= 9)	5 (62.5)	9 (60)	1 (50)	5 (55.6)	4 (80)
1–2.5 (n = 4)	2 (25)	3 (20)	0	3 (33.3)	1 (20)
2.6–10 (n = 1)	0	1 (6.7)	0	0	0
>10 (n = 2)	1 (12.5)	2 (13.3)	1 (50)	1 (11.1)	0
*Statistics*	χ^2^ = 0.955 *p* = 0.812	χ^2^ = 1.942 *p* = 0.585	χ^2^ = 3.517 *p* = 0.319	χ^2^ = 1.348 *p* = 0.718	χ^2^ = 2.030 *p* = 0.566
7. Horses are always maintained outside					
Yes (n = 8)	4 (50)	8 (53.3)	2 (100)	4 (44.4)	1 (20)
Depending on the weather (n = 4)	2 (25)	4 (26.7)	0	3 (33.3)	3 (60)
No (n = 4)	2 (25)	3 (20)	0	2 (22.2)	1 (20)
*Statistics*	χ^2^ = 0.470 *p* = 0.791	χ^2^ = 3.356 *p* = 0.187	χ^2^ = 2.133 *p* = 0.344	χ^2^ = 0.682 *p* = 0.711	χ^2^ = 4.636 *p* = 0.098
8. Horses can graze daily					
Yes (n = 10)	5 (62.5)	10 (66.7)	2 (100)	5 (55.6)	2 (40)
No (n = 6)	3 (37.5)	5 (33.3)	0	4 (44.4)	3 (60)
*Statistics*	χ^2^ = 0.282 *p* = 0.596	χ^2^ = 1.155 *p* = 0.282	χ^2^ = 1.280 *p* = 0.258	χ^2^ = 0.121 *p* = 0.728	χ^2^ = 1.473 *p* = 0.225
9. Horses traveling outside the center					
Yes (n = 9)	4 (50)	8 (53.3)	2 (100)	5 (55.6)	2 (40)
No (n = 7)	4 (50)	7 (46.7)	0	4 (44.4)	3 (60)
*Statistics*	χ^2^ = 0.119 *p* = 0.730	χ^2^ = 4.468 *p* = 0.031 *	χ^2^ = 1.659 *p* = 0.198	χ^2^ = 0.029 *p* = 0.865	χ^2^ = 0.732 *p* = 0.392
10. Automated horse waterers					
Yes (n = 10)	3 (37.5)	9 (60)	2 (100)	4 (44.4)	3 (60)
No (n = 6)	5 (62.5)	6 (40)	0	5 (55.6)	2 (40)
*Statistics*	χ^2^ = 4.001 *p* = 0.046 *	χ^2^ = 0.601 *p* = 0.439	χ^2^ = 1.286 *p* = 0.257	χ^2^ = 2.683 *p* = 0.101	χ^2^ = 0.018 *p* = 0.893
11. Presence of manure near horse boxes					
Yes (n = 9)	4 (50)	9 (60)	1 (50)	5 (55.6)	4 (80)
No (n = 7)	4 (50)	6 (40)	1 (50)	4 (44.4)	1 (20)
*Statistics*	χ^2^ = 0.560 *p* = 0.454	χ^2^ = 0.166 *p* = 0.683	χ^2^ = 0.076 *p* = 0.783	χ^2^ = 0.462 *p* = 0.497	χ^2^ = 1.563 *p* = 0.211
12. Observation of Diptera during winter					
Yes (n = 6)	3 (37.5)	5 (33.3)	0	4 (44.4)	3 (60)
No (n = 10)	5 (62.5)	10 (66.7)	2 (100)	5 (55.6)	2 (40)
*Statistics*	χ^2^ = 0.003 *p* = 0.953	χ^2^ = 0.057 *p* = 0.811	χ^2^ = 1.280 *p* = 0.258	χ^2^ = 0.030 *p* = 0.862	χ^2^ = 1.473 *p* = 0.225
13. Confirmed cases of allergy to mosquitoes					
Yes (n = 5)	4 (50)	5 (33.3)	0	5 (55.6)	3 (60)
No (n = 11)	4 (50)	10 (66.7)	2 (100)	4 (44.4)	2 (40)
*Statistics*	χ^2^ = 1.837 *p* = 0.175	χ^2^ = 0.016 *p* = 0.901	χ^2^ = 0.970 *p* = 0.325	χ^2^ = 2.116 *p* = 0.146	χ^2^ = 2.623 *p* = 0.105
14. Application of some measures for the control of insects					
Yes (n = 2)	1 (12.5)	2 (13.3)	0	2 (22.2)	1 (20)
No (n = 14)	7 (87.5)	13 (86.7)	2 (100)	7 (77.8)	4 (80)
*Statistics*	χ^2^ = 0.067 *p* = 0.796	χ^2^ = 0.031 *p* = 0.861	χ^2^ = 0.305 *p* = 0.581	χ^2^ = 0.260 *p* = 0.610	χ^2^ = 0.351 *p* = 0.554

* Statistically significant (*p ≤* 0.05).

## Data Availability

Data presented in this study are available upon request from the corresponding author.

## Abbreviations

The following abbreviations are used in this manuscript:

*An.*	*Anopheles*
*Ae.*	*Aedes*
*C.*	*Culicoides*
CDC-UV	Center for Diseases Control and Prevention-Ultraviolet Traps
*Cs.*	*Culiseta*
*Cx.*	*Culex*
*Oc.*	*Ochlerotatus*
Km	Kilometers
NW	Northwest
UV	Ultraviolet
